# Dr. Ellwood on Medical Gardening

**Published:** 1914-11-07

**Authors:** 


### Dr. Ellwood on Medical Gardening.

### PUBLIC PHARMACISTS' AND DISPENSERS' ASSOCIATION.

The Public Pharmacists' and Dispensers' Association opened its winter session on October 28, and, despite the absence of several members on active service, there was a good attendance. Mr. Peter MacEwan, F.C.S., was in the chair, others present being Messrs. T. H. W. Idris, J.P., A. P. Atkinson, R. W. Lindsey, France, Bullen, Gibson, Miller, Jenkin, Goodall, Skinner, Tocher; Misses Bedell, Tufnell, Davidson, Cutfield, etc.

Dr. T. Ashcroft Ellwood delivered a lecture on "The Influence of the War on Medicine and Pharmacy." Describing the immediate effect, the lecturer showed that it had been to call out for service a large number of qualified medical men and pharmacists. The deficiency thus caused would require to be made up by loyalty and harder work on the part of those remaining. We have been very dependent on foreign countries for our supply not only of fine chemicals, but of roots, leaves, etc. British manufacturers were waking up and now already were manufacturing a great number of these chemicals. Dr. Ellwood suggested that we should induce the farmers to go in for drug cultivation, incidentally mentioning that he grew some twenty-five medicinal plants for professional use.

The remote effect of the war would be a return to civil life of a large number of doctors and pharmacists. Dr. Ellwood made some pertinent remarks on the loyalty of those authorities who keep posts open, and in view of improved trade after the war no one need be ousted from his post. We should encourage research, get duty-free alcohol for all manufactures, and make a bid for Continental custom for our drug manufactures.

Mr. MacEwan, dealing with our position regarding drugs and chemicals, mentioned that the most important drug required at the Front was morphia, and for this Germany was dependent on supplies obtained from England. Messrs. Idris, Atkinson, Bullen, France, and others joined in a useful discussion which embraced Army rank for pharmacists, cultivation of drugs, trade names, and treatment for dependants.

